# Population versus hospital controls for case-control studies on cancers in Chinese hospitals

**DOI:** 10.1186/1471-2288-11-167

**Published:** 2011-12-15

**Authors:** Lin Li, Min Zhang, D'Arcy Holman

**Affiliations:** 1 School of Population Health, The University of Western Australia, 35 Stirling Highway, Crawley, WA 6009, Australia

## Abstract

**Background:**

Correct control selection is crucial to the internal validity of case-control studies. Little information exists on differences between population and hospital controls in case-control studies on cancers in Chinese hospital setting.

**Methods:**

We conducted three parallel case-control studies on leukemia, breast and colorectal cancers in China between 2009 and 2010, using population and hospital controls to separately match 540 incident cases by age, gender and residency at a 1:1 ratio. Demographic and lifestyle factors were measured using a validated questionnaire in face-to-face interview. Odds ratios (ORs) and 95% confidence intervals (CIs) were obtained using conditional logistic regression analyses.

**Results:**

The two control groups had closely similar exposure distributions of 15 out of 16 factors, with the only exception being that hospital controls were less likely to have a BMI ≥ 25 (OR = 0.71, 95% CI: 0.54, 0.93). For exposure of green tea drinking, the adjusted ORs (95% CIs) comparing green tealeaves intake ≥ 1000 grams annually with non-drinkers were 0.51 (0.31, 0.83) and 0.21 (0.27, 0.74) for three cancers combined, 0.06 (0.01, 0.61) and 0.07 (0.01, 0.47) for breast cancer, 0.52 (0.29, 0.94) and 0.45 (0.25, 0.82) for colorectal cancer, 0.65 (0.08, 5.63) and 0.57 (0.07, 4.79) for leukemia using hospital and population controls respectively.

**Conclusions:**

The study found that hospital controls were comparable with population controls for most demographic characteristics and lifestyle factors measured, but there was a slight difference between the two control groups. Hospital outpatients provide a satisfactory control group in hospital-based case-control study in the Chinese hospital setting.

## Background

Correct control selection is crucial to the internal validity of case-control studies [1,2]. The function of controls is to provide valid information on the distribution of exposure within the population at risk of becoming a case [1]. When cases are ascertained from hospitals serving a defined geographic area, a probability sample of unaffected individuals from the population of that geographic region can be used to enhance the likelihood that cases and controls come from the same source population. A concern with this approach is that population controls sampled from a convenient population register may not be representative of the true population at risk of being a member of a hospital case series; population controls may also lack comparability in recruitment fraction and recall of information [1-5]. But there are also concerns that hospital controls, especially those with other diseases, may fail to provide an unbiased sample of the population at risk with respect to exposure status [1-5].

When we applied successfully to Australia's National Health and Medical Research Council to perform case-control studies in China on the effects of green tea on the incidence rates of colorectal cancer, breast cancer and adult leukemia, it became a condition of funding that we recruit both hospital and population controls for around one fifth of the case series to determine if there was any difference of practical importance.

A handful of studies have compared different control groups in western countries where non-emergency access to hospital care generally depends on referral from medical practitioners in non-institutional settings [6-14]. When researchers have enrolled both hospital and population controls, differences in effect estimates using the two groups have varied from inconsequential [6,7] to problematic [8-10], prompting discussions about characteristics of the exposure, selection protocols and clinical dynamics. Some studies have specifically reported differences between hospital and population controls in key exposure distributions [11-14], but elsewhere the two control groups were found to have very similar exposure distributions [15].

It was important to understand whether exposures measured in the study were in agreement when ascertained from population and hospital control groups, especially because our previous case-control studies of green tea and cancer in China had used hospital controls. This study aimed, therefore, to compare key exposure distributions between hospital controls and alternative population controls to determine if there was any difference of practical importance, and to examine if inferences drawn from green tea effect estimates for different cancer case groups using hospital controls would be different from those using population controls in the Chinese hospital setting.

## Methods

### Study design and participants

The study, as a validation component of three large case-control studies of malignancies, was conducted in Shenyang, the capital city of Liaoning Province, Northeast China. In August 2009 to July 2010, 540 incident cases with a primary diagnosis of leukemia, or breast cancer, or colorectal cancer were identified from histopathology and haematology records at the First Hospital of China Medical University, a public teaching hospital with 2,249 beds, around 32,000 inpatients annually and 3,000 outpatients daily. The eligible cases were permanent residents of urban Shenyang aged 18 to 85 years. During the study period, 540 population controls and 540 hospital controls were selected from Shenyang residents and from outpatients at the same hospital to separately match cases in a 1:1 ratio. Most of controls were interviewed within three months after cases were interviewed.

The methods of recruiting population controls were similar to those used in case-control studies in Shanghai, China [16,17]. Population household registries, which kept records of all permanent residents in urban Shenyang, were used to select controls from the five metropolitan districts in Shenyang, namely Heping, Shenhe, Dadong, Huanggu, and Tiexi. With the assistance of the local community councils, residents who lived at their registered address during the study period were randomly selected from household registry rolls. Residents were eligible as population controls if they matched with individual cases of an updated list by gender and year-of-birth quinquennium on a given selection day.

Hospital controls were drawn from the population of patients in the hospital [18]. A systematic selection process used in our previous studies was adopted for hospital control recruitment [19]. They were selected from outpatients who attended the Medical Examination Centre at the First Hospital of China Medical University and were permanent residents of urban Shenyang. The eligible hospital controls were those without any malignancy after they had consulted their doctors. Each hospital control was selected as the first attendee on a given selection day to match the next case on a daily updated list of cases by sex and 5-year age group. Hospital outpatients were excluded as a control if they were not matched their corresponding cases by gender, year-of-birth quinquennium and living areas, and if they had a diagnosis of any malignancy before or after recruitment. The project protocol had received ethics approval from both the Human Research Ethics Committee of The University of Western Australia and the First Hospital of China Medical University authority.

### Questionnaire and interview

Subjects were briefed regarding confidentiality and anonymity issues and the general aims of the study to investigate lifestyle factors. An appointment for an interview was made after obtaining the respondent's consent via an initial contact. A face-to-face interview was then conducted by the first author, using a structured questionnaire and usually took 30-40 minutes. The validated questionnaire, available from the authors upon request, was used to collect the information on: (i) demographic and lifestyle characteristics, e.g., area of residence, education, smoking, alcohol and tea consumption and physical activity; (ii) dietary intake assessed by a food frequency questionnaire (FFQ); and (iii) factors relevant to hormonal status and family history of cancer. The questionnaire was adapted from that used in our previous studies on cancers [20]. This instrument was originally modified from one used for studying cancers in Shanghai in order to ensure cultural relevance [21]. The questionnaire was translated into Chinese and checked using back-translation by professional Chinese translators. The internal consistency and reliability of the questionnaire was assessed in a preliminary study and then evaluated by a test-retest. The intraclass correlation coefficients for mean daily intake of tea and alcohol were 0.83 and 0.88 [22]. Thus high coefficients for test-retest reliability suggested that the questionnaire may be relied upon in assessing selected demographic characteristics and lifestyle factors [22].

### Statistical analysis

All data were checked at the end of each interview for completeness and were coded and analysed using SPSS version 18.0. Participants' self-reported current height in meters and weight in kilograms were used to calculate body mass index (BMI) (weight/height^2^). Daily energy intake and alcohol consumption were assessed using the FFQ. The frequencies of 100 food items, including beer, wine, and liquor intake, were assigned into nine categories: never or hardly ever; once a month; 2-3 times a month; once a week; 2-3 times a week; 4-6 times a week; once a day; twice a day; and ≥ 3 times a day. Food and alcohol consumption was based on habitual diet and a 'reference' recall period was set as one year prior to diagnosis for cases or interview for controls. If there was any recent change in habits, only information on the habits before the change was used in data analysis. The frequency and quantity variables derived from the FFQ were converted into daily food consumption, adjusted for the edible portions of foods, cooking methods, seasonal factors, and market availability [23]. Total energy intake was estimated using Chinese Food Composition Tables [24]. The frequency and quantity variables for beer, wine, and liquor were converted into daily intake in ml. Amounts of ethanol ingested were calculated by assuming 10 g of ethanol per 285 ml of beer, per 100 ml of wine, and per 30 ml of liquor based on a method used in a previous study [25]. Physical activity was expressed in terms of weekly metabolic equivalent task hours (MET hrs/week) [26]. MET scores of 6, 4.5, and 2.5 were assigned respectively for vigorous, moderate, and walking activity based on a compendium of physical activities [27]. The quantitative variables for physical activity (MET) were divided into quartiles based on the corresponding empirical distribution of population controls, with the lowest quartile being the reference category.

Selected demographic characteristics and lifestyle factors in both control groups were first compared using a t-test for continuous variables and chi-square test for categorical variables. Associations of hospital/population control status with exposure variable were then assessed using adjusted odds ratios (ORs), 95% confidence intervals (95% CI) and p-values for trend estimated from conditional logistic regression adjusted for education, income, household size, BMI, smoking, alcohol, tea consumption, energy intake (kcal), and physical activity. The ORs were for the odds of being a hospital rather than population control. Each quantitative or ordinal quantitative measure was subjected to a linear trend test.

All cancer case groups combined, breast cancer, colorectal cancer, and leukemia only subgroups were analysed separately using the two types of matched control groups with regard to green tea intake using conditional logistic regression, adjusted for education, BMI 5 years ago, smoking, passive smoking, alcohol consumption, energy intake (kcal), physical activity, and cancer in first degree relative. For breast cancer only subgroup, the regressions analyses also adjusted for menopausal status, oral contraceptive use, and number of children breastfed.

## Results

Data were collected on a total of 1080 controls. Interviews were completed for 540 (89.7%) of the 602 eligible population controls who were approached to participate. The only reason for non-participation was refusal to provide a blood sample, which was an essential part of the study. Of 563 eligible hospital controls who were approached to participate, interviews were completed also for 540 (95.9%). For the 540 recruited cancer cases, the response fraction was 98.5%.

Comparisons of distributions of demographic characteristics and lifestyle factors between hospital and population controls are shown in Table 1. The two control groups were remarkably similar in their exposure distributions for age (forced by matching), marital status, household size, education, income, body mass index, smoking, alcohol intake, physical activity, tea drinking and family history of malignancy.

**Table 1:** Comparison of demographic characteristics and lifestyle factors between hospital and population controls

Factor	Hospital controls (n = 540)	Population controls (n = 540)	***P***_**value**_^**a**^
Age (years)	56.2 (10.6)	56.2 (10.6)	
Marital status			0.64
Married	499 (92.4)	504 (93.3)	
Others	41 (7.6)	36 (6.7)	
Household size	2.9 (1.1)	3.0 (1.0)	0.20
Education			0.50
No or primary school	46 (8.5)	37 (6.9)	
Junior high school	208 (38.5)	219 (40.6)	
Senior high school	119 (22.0)	106 (19.6)	
Tertiary education	167 (30.9)	178 (33.0)	
Income (per capita, Yuan/month)			0.50
≤ 1000	69 (12.8)	80 (14.8)	
1001-2000	347 (64.3)	330 (61.1)	
≥ 2001	124 (23.0)	130 (24.1)	
BMI (kg/m^2^)	23.7 (3.1)	24.0 (3.3)	0.07
Smoking (≥ 20 packs in lifetime)	98 (18.1)	104 (19.3)	0.70
Years of smoking	5.2 (11.9)	5.1 (11.4)	0.86
Number of cigarettes per day	3.2 (7.7)	3.4 (8.2)	0.59
Quitting smoking	20 (3.7)	12 (2.2)	0.21
Alcohol consumption (ethanol g/day)	6.6 (20.8)	5.6 (16.0)	0.39
Years of green tea drinking	5.1 (9.7)	5.5 (10.2)	0.47
Dried green tea leaves (g/year)	251.0 (539.7)	282.1 (665.8)	0.40
Physical activity (MET hrs/week)	56.4 (49.0)	53.9 (42.8)	0.36
Malignancies in first degree relatives	81 (15.0)	90 (16.7)	0.53

Values expressed as mean (SD) or number (percent).

Abbreviations: BMI, body mass index; MET, metabolic equivalent tasks.

^a ^Two-sided *t*-test for continuous variables and chi-square test for categorical variables.

Table 2 presents the adjusted ORs for demographic characteristics and lifestyle factors, representing the tendency for each factor to associate independently with status as a hospital rather than population control. The 29 points estimates of non-baseline ORs ranged from 0.46 to 1.55, whilst 19 were within the range of 0.80-1.25. Two of the 29 confidence intervals excluded the null value, being associated with low ORs for a household size of 3 members compared with 1-2 members (0.46) and a BMI ≥ 25 compared with < 25 (0.71). However, there was no consistent trend in the association of household size with hospital/population control status, the OR for a household size of ≥ 4 compared with 1-2 members being 0.83 (p-value for trend = 0.21). For the key important study exposure of green tea drinking frequency, the ORs revealed no evidence of association with control type, being 0.94 at ≤ 6 times a week and 1.08 at ≥ 1 time per day compared with never or seldom drinking green tea (p-value for trend = 0.58).

**Table 2:** Adjusted OR for the odds of being a hospital control according to demographic, lifestyle factors

Characteristics	No. hospital/population controls	Adjusted OR^a ^(95% CI)
Education		
No or primary school	46/37	1.00^b^
Junior high school	208/219	0.65 (0.38, 1.11)
Senior high school	119/106	0.80 (0.45, 1.41)
Tertiary education	167/178	0.65 (0.37, 1.14)
Income (per capita, Yuan/month)		
≤ 1000	69/80	1.00
1001-2000	347/330	1.50 (0.96, 2.33)
≥ 2001	124/130	1.54 (0.91, 2.61)
Marital status		
Married	499/504	1.00
Others	41/36	1.13 (0.66, 1.94)
Household size		
1-2	209/158	1.00
3	220/288	0.46 (0.32, 0.65)
≥ 4	111/94	0.83 (0.58, 1.19)
BMI (kg/m^2^)		
< 25	387/349	1.00
≥ 25	153/191	0.71 (0.54, 0.93)
Malignancies in first degree relatives		
No	459/450	1.00
Yes	81/90	0.84 (0.59, 1.17)
Smoking (≥ 20 packs in lifetime)		
No	442/436	1.00
Yes	98/104	0.86 (0.58, 1.28)
Years of smoking		
0	442/436	1.00
≤ 20	29/38	0.69 (0.40, 1.20)
> 20	69/66	1.00 (0.62, 1.63)
Number of cigarettes per day		
0	442/436	1.00
≤ 10	33/40	0.73 (0.42, 1.26)
> 10	65/64	0.95 (0.60, 1.50)
Quitting smoking		
No	520/528	1.00
Yes	20/12	1.55 (0.74, 3.24)
Alcohol consumption		
No	347/338	1.00
Yes	193/202	0.92 (0.68, 1.24)
Alcohol consumption (ethanol g/day)		
0	347/338	1.00
≤ 15	140/145	0.91 (0.66, 1.25)
> 15	53/57	0.94 (0.56, 1.58)
Green tea drinking frequency		
Never or seldom	363/362	1.00
≤ 6 times a week	53/58	0.94 (0.61, 1.43)
≥ 1 times per day	124/120	1.08 (0.79, 1.48)
Years of green tea drinking		
0	363/362	1.00
≤ 10	92/91	1.05 (0.75, 1.49)
> 10	85/87	1.00 (0.69, 1.46)
Dried green tea leaves (g/year)		
0	363/362	1.00
< 500	52/61	0.81 (0.52, 1.25)
500- < 1000	66/50	1.45 (0.95, 2.22)
≥ 1000	59/67	0.88 (0.58, 1.34)
Physical activity (MET hrs/week)		
≤ 27.42	132/135	1.00
> 27.42-43.75	126/135	0.97 (0.68, 1.39)
> 43.75-73.25	158/135	1.22 (0.86, 1.73)
> 73.25	124/135	1.01 (0.69, 1.47)

Abbreviations: MET, metabolic equivalent tasks; OR, odds ratio; CI, confidence interval.

^a ^Estimates from conditional logistic regression models included terms for education (no or primary school, junior high school, senior high school, tertiary education), income (per capita, ≤ 1000, 1001-2000, ≥ 2001 Yuan/month), household size (continuous), BMI now (continuous), smoking (no, yes; not for smoking variables), alcohol consumption (no, yes; not for alcohol variables), tea drinking (no, yes; not for tea variables), physical activity (weekly MET-hours, continuous), kilocalories (continuous).

^b ^Reference group.

Table 3 presents ORs according to green tea intake for the cancers combined and for each cancer case subgroups, using the two types of matched control groups. All ORs for the frequency of green tea drinking and amount of dried green tea leaves consumed (g/year) were on the same side of the null value and quite close in all the comparisons estimated using the 2 types of control groups. In the final models, compared with nondrinkers vs. dried green tealeaves intake ≥ 1000 grams annually, the adjusted ORs (95% CI) were 0.51 (0.31, 0.83) and 0.21 (0.27, 0.74) for all cancer case groups combined; 0.06 (0.01, 0.61) and 0.07 (0.01, 0.47) for breast cancer; 0.52 (0.29, 0.94) and 0.45 (0.25, 0.82) for colorectal cancer; 0.65 (0.08, 5.63) and 0.57 (0.07, 4.79) for leukemia using hospital outpatient and population controls respectively in each instance.

**Table 3:** Adjusted odds ratios for green tea intake by hospital and population controls

	No. cases/hospital controls	Adjusted OR^a ^(95% CI)	No. cases/population controls	Adjusted OR^a ^(95% CI)
*All cancer cases*				
Frequency of green tea drinking				
Never or seldom	353/363	1.00^b^	353/362	1.00
≥ 1 times per day	72/124	0.77 (0.59, 1.00)	72/120	0.78 (0.60, 1.01)
Dried green tea leaves (g/year)				
0	353/363	1.00	353/362	1.00
500- < 1000	24/66	0.60 (0.40, 0.92)	24/50	0.71 (0.47, 1.08)
≥ 1000	17/59	0.51 (0.31, 0.83)	17/67	0.21 (0.27, 0.74)
*Breast cancer only^c^*				
Frequency of green tea drinking				
Never or seldom	199/211	1.00	199/226	1.00
≥ 1 times per day	25/49	0.55 (0.28, 1.06)	25/37	0.61 (0.32, 1.18)
Dried green tea leaves (g/year)				
0	199/211	1.00	199/226	1.00
500- < 1000	14/28	0.54 (0.22, 1.33)	14/20	0.60 (0.24, 1.50)
≥ 1000	3/16	0.06 (0.01, 0.61)	3/15	0.07 (0.01, 0.47)
*Colorectal cancer only*				
Frequency of green tea drinking				
Never or seldom	140/132	1.00	140/111	1.00
≥ 1 times per day	35/65	0.67 (0.45, 0.99)	35/72	0.62 (0.42, 0.92)
Dried green tea leaves (g/year)				
0	140/132	1.00	140/111	1.00
500- < 1000	10/29	0.51 (0.26, 0.99)	10/27	0.55 (0.28, 1.06)
≥ 1000	13/39	0.52 (0.29, 0.94)	13/46	0.45 (0.25, 0.82)
*Leukemia only*				
Frequency of green tea drinking				
Never or seldom	14/20	1.00	14/25	1.00
≥ 1 times per day	12/10	1.46 (0.55, 3.84)	12/11	1.55 (0.61, 3.96)
Dried green tea leaves (g/year)				
0	14/20	1.00	14/25	1.00
500- < 1000	0/9	-	0/3	-
≥ 1000	1/4	0.65 (0.08, 5.63)	1/6	0.57 (0.07, 4.79)

^a ^Estimates from conditional logistic regression models included terms for education (no or primary school, junior high school, senior high school, tertiary education), BMI 5 years ago (continuous), smoking (no, yes), passive smoking (no, yes), alcohol consumption (no, yes), physical activity (weekly MET-hours, continuous), energy intake (continuous, kilocalories), cancer in first degree relative.

^b ^Reference group.

^c ^Models also included terms for menopausal status, oral contraceptive use, and number of children breastfed.

## Discussion

This validation study, as a component of three large case-control studies of malignancies, used separate hospital outpatient and population control groups to assess differences in the distributions of demographic characteristics and lifestyle factors between the two control groups. Furthermore, the study evaluated if different conclusions were reached when comparing cases with matched hospital controls or population controls in the effect of green tea intake. The study found that the distributions of study exposures in hospital outpatient controls were similar to those in population controls. In addition, we obtained similar results in assessing the effect of green tea drinking on three cancers together and separated, using hospital outpatient controls and population controls respectively. Therefore, our results suggest that in the context of a case-control study conducted at a Chinese hospital using urban cases, regardless of whether controls were selected from hospital outpatient attendees without malignancy or drawn from population household registries covering populations in the catchment area of the participating hospital, the two control groups appeared to have similar exposure distributions. Our results identified one exception to this general conclusion. It is possible that population controls may tend to report higher levels of being overweight than hospital outpatient controls.

The findings of this investigation were consistent with a smaller pilot study conducted in 1999-2000 by our research team [20], and with other studies which suggested that population and hospital controls were generally similar in their studies [8-10], as well as with a research by other investigators, who compared non-cancer outpatients with population controls in a case-control study in Japan [15].

A key exposure in our work was green tea drinking measured in three parallel case-control studies. For three cancers together, breast cancer, colorectal cancer, and leukemia, the inversed associations were similar in their direction and size regardless of which control series was employed to represent the underlying exposure to higher green tea consumption. The results, using either type of control series, were consistent with our previous finding that increasing the quantity of green tea consumption reduces the risk of breast cancer in Chinese women [19], as well as the results from four studies pooled in a meta-analysis, which reported a reduced risk of breast cancer for highest versus non/lowest intake of green tea [28]. The association between colorectal cancer and green tea consumption assessed in this study were consistent with another meta-analysis combining results from eight studies for high green tea intake [29]. Although we recruited less leukemia cases, an inverse association was also observed, although it fell short of statistical significance, which was similar to our previous study [30]. The effects of green tea consumption estimated in this study suggest that hospital outpatient controls performed little different from population controls in green tea exposures.

Whilst hospital outpatient and population controls selected in this study may function in a similar manner for a hospital case-control design in China, this does not entirely resolve the underlying theoretical debate as to which of the two types of control group is the more defensible. For BMI, for example, where our study did find evidence of some practical and statistical differences, one is left with the question of which control exposure distribution is more representative of that in the population at risk of becoming a case. The result may have simply been due to chance, although the possibility that population controls were more likely to be overweight cannot be ignored. On the other hand, the lower response fraction of population controls (89.7% vs 95.9% for hospital controls) may have provided the circumstances where normal weight individuals in the general population were less inclined to participate in a research study on health and lifestyle.

In China, public hospitals provide treatment services for acute, severe, and critical outpatients and inpatients, and they fulfill education, research, prevention, and rehabilitation objectives by providing special medical services. All public hospitals have preventive health care branches, which are responsible for reporting infectious diseases, health checks, health counseling, community prevention services, health education, disease screening, family planning and birthing guidance, and care of staff [31]. Patients living in cities readily visit hospitals as non-referred outpatients for check-ups. Cultural factors and insurance arrangements lead patients to maintain a strong relationship with one particular hospital, where they receive a complete range of health care [32,33]. Survey data in 2008 showed low levels (14%) of community health service utilization, suggesting that community health services are not yet the first point of contact with the health system in China [34]. Therefore, hospital outpatients in China are somewhat similar to ambulatory patients visiting GP clinics in Western countries.

As there is almost never one ideal control group, some researchers believe that population registers provide the most valid way of sampling controls in a hospital case-control study [5,11-14], because the main theoretical strength of population controls is the potential to provide information of exposure that is unaltered by associations with illness [1]. Others suggest that generally, hospital controls should be preferred in a hospital case-control study regarding practicability, cost and travel time for face to face interviews [9,10,15]. There may also be differences in the capacity to recall and report exposures between randomly selected population members and those who are actively engaged in the health system [1]. In addition, only hospital controls have shown some evidence that in the event of developing the cancer, they would be likely to attend the hospital and become a case in the study [1-3].

## Conclusion

With respect to the demographic characteristics and lifestyle factors included in the present study, our results suggest that hospital outpatient controls performed little different from population controls for most exposures. For the key exposure, the effect estimates for different cancer case groups using hospital outpatient controls were similar to those using population controls. Therefore, even though some points of concern exist, hospital outpatients provide a satisfactory control group in hospital-based case-control study in the Chinese hospital setting.

## List of abbreviations

MET-hour: metabolic equivalent task hour; BMI: Body mass index; OR: odds ratio; 95% CI: 95 percent confidence interval.

## Competing interests

The authors declare that they have no competing interests.

## Authors' contributions

LL collected the data, conducted data analysis, interpretation, and drafted the manuscript. MZ designed the study, directed all aspects of its implementation, including quality assurance and control, assisted data analysis, and reviewed drafts of the article. DH designed the study, supervised all aspects of its implementation, and reviewed drafts of the article. All authors have read and approved the final manuscript.

## Pre-publication history

The pre-publication history for this paper can be accessed here:

http://www.biomedcentral.com/1471-2288/11/167/prepub
